# Novel Diagnostic Algorithm for the Floating and Sunken Pulse Qualities and Its Clinical Test

**DOI:** 10.1155/2011/813427

**Published:** 2011-03-01

**Authors:** Jaeuk U. Kim, Young Ju Jeon, Yu Jung Lee, Keun Ho Kim, Jong Yeol Kim

**Affiliations:** Division of Constitutional Medicine Research, Korea Institute of Oriental Medicine, Daejeon 305-811, Republic of Korea

## Abstract

We propose a novel classification algorithm for the floating pulse and the sunken pulse using a newly defined coefficient (*C*_fs_). To examine the validity of the proposed algorithm, we carried out a clinical test in which 12 oriental medical doctors made pairwise diagnoses on the pulses of volunteering subjects. 169 subjects were simultaneously diagnosed by paired doctors, and the diagnoses in 121 subjects were concordant, yielding an accuracy of 72% and a Matthews correlation coefficient of 0.42, which indicates reasonable agreement between doctors. Two sample *T*-tests showed that subjects in the sunken pulse group had significantly higher BMI and *C*_fs_ (*P* < .05) than those in the floating pulse group. The pulse classification by the algorithm converged with the diagnoses of paired doctors with an accuracy up to 69%. With these results, we confirm the validity of the novel classification algorithm for the floating and sunken pulses.

## 1. Introduction

Pulse diagnosis is one of the four important diagnostic methods in oriental medicine, which are inspection, listening and smelling, inquiring, and palpation [1–4]. The primary site for pulse diagnosis is the radial artery placed on both wrists. To make a pulse diagnosis, oriental medical doctors (OMDs) divide the terminal region of the radial artery into three adjacent intervals of Chon, Gwan, and Cheok, and use index, middle, and ring fingers on each palpating position. By pressing with these fingers with varying pressure simultaneously or sequentially, doctors determine characteristic pulse qualities. By analyzing the pulse qualities, primarily, doctors obtain information on balance in health and illness or subtle changes in homeostasis [3]. 

Pulse diagnosis requires sophisticated palpation techniques and interpretation on the pulse qualities demands enduring efforts with many clinical trials and case studies. For this reason, experiential bias and subjective judgments remain difficult to avoid. To overcome technical difficulties, to answer doubts and skepticism on its clinical validity, and to go beyond its current state of the art, it is necessary to develop objective techniques for pulse-taking using standardized protocols and quantitative definitions of the pulse qualities in terms of a few measureable physical parameters [5–7].

To quantify and standardize pulse diagnosis, it is essential to develop palpation devices that provide objective pulse signals as a function of the hold-down pressure, and to develop classification algorithms of the pulse qualities by analyzing the measured pulses. At the same time, the validity and efficiency of pulse diagnosis need to be verified clinically by objective methods. Thus far, some progress has been made on the quantification and standardization of pulse diagnosis. For example, pulse devices equipped with arrays of piezoresistive pressure sensors have been developed. In addition, clinical data have been collected by pulse devices and it has been analyzed by statistical methods. Studies on the fundamental aspects such as the blood flow dynamics along the radial artery and classifications of pulse waveforms are currently under investigation [8–18]. 

Pulse-taking devices should be able to measure pulse waveforms for several different steps of hold-down pressure, and it should be able to record the spatial distribution of the pulse strength along the axis of the radial artery and in its cross-section [5]. With a pulse-taking device which supplies the above-described quantities with guaranteed intrarater reliability (repeatability) and interrater reliability (reproducibility), we can obtain some principal characteristics of the pulse such as the level of the depth, width, length, and strength of the pulse, as well as the speed or rate of the pulse. As there are some literature studies which reinterpreted the pulse qualities in the classic textbooks in terms of these measurable quantities [6, 7], a desirable direction to develop classification algorithms for the pulse qualities is to understand a pulse by simple physical quantities. 

For developing a reliable classification algorithm for the pulse qualities, the algorithm needs to be validated by a clinical test with a relevant protocol. The pulse-taking device used should have guaranteed repeatability and reproducibility, and the pulse qualities determined by the algorithm should be tested for consistency with the OMDs' diagnoses. As a first step towards developing reliable classification algorithms, in this work, we introduce a novel classification algorithm for the floating and sunken pulses. To study the validity of the suggested algorithm, we study clinically the correlation between algorithmic outcomes and the OMDs' diagnoses. The floating and sunken pulses belong to four principal pulses together with the rapid and slow pulses [1]. Therefore, one cannot emphasize enough the importance of developing a reliable classification algorithm for the floating and sunken pulses.

## 2. Quantification of the Floating and Sunken Pulses

### 2.1. Floating Pulse and Sunken Pulse

According to “Mai Jing”, the floating pulse is a pulse potent when felt with no pressure applied but impotent when felt with pressure applied, and the sunken pulse is a pulse impotent when felt with no pressure applied but potent when felt with pressure applied, while the hidden pulse is a pulse imperceptible until the fingers touch the bone with extremely heavy pressure [3]. Similar definitions can be found in other classics such as “Bin Hue Mai Xue” or “Yi Xue Ru Men”. A brief hypothetical explanation for the characteristic features of the floating and sunken pulses is as follows. The floating pulse is characterized as a Yang pulse and it is mostly seen in wind evil, external guest, and exterior patterns. The sunken pulse, on the other hand, is characterized as a Yin pulse and it is largely seen in those who have pathologic Qi hidden internally or interior patterns [2]. In general, superficial pulses are associated with acute diseases of exterior origin, while deep pulses are indicative of more severe and chronic illnesses related to the internal organs. For instance, the sunken pulse without other symptoms indicates a high potential for the impaired immune system. It is worth noting that the pulses of obese subjects are normally deeper than those of thin individuals [4].

### 2.2. Coefficient of the Floating and Sunken Pulses

**Figure 1:**
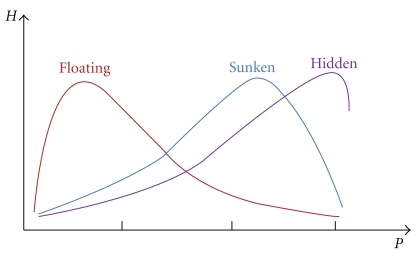
Floating versus sunken versus hidden pulses distinguished in the *P*-*H* curve [6].

There have been suggestions on how to quantify the pulse qualities defined only by the level of depth using pulse signals obtained by pulse-taking devices. As expounded by Fei [6], the floating/sunken/hidden pulses are distinguishable from the shape of the *P*-*H* curve (the curve of the pulse strength (*H*) as a function of the hold-down pressure (*P*)) as shown in Figure 1. Indeed, there are some pulse qualities other than the floating or sunken pulses that appear either at the superficial level or deep level, and they can be categorized as floating-type pulses or sunken-type pulses [1]. For instance, the scallion stalk pulse is a pulse exhibiting a floating characteristic combined with large but soft features (empty in the middle but solid at the sides when pressure is applied) [3]. In this aspect, strictly speaking, the *P*-*H* curve alone is insufficient to distinguish the floating or sunken pulses from more complex pulses. However, for the sake of simplicity, in this work, we do not distinguish the floating and sunken pulses from floating-type and sunken-type pulses and suppose them as the floating and sunken pulses. As shown in Figure 1, the hidden pulse can be considered the extreme of the sunken pulse and we will only focus on the floating and sunken pulses. 

**Figure 2:**
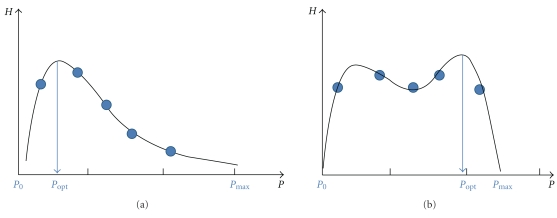
*P*-*H* curve with interpolation (solid curve) for a few discrete data points (circles) for the calculation of *C*_fs_ [19]. Examples for (a) a floating pulse, (b) an erroneous decision as a sunken pulse. Compare (b) with Figure 3(b).

Utilizing the idea in Figure 1, Lee et al. introduced the coefficient of the floating and sunken pulses (*C*_fs_) defined by [19]

(1) $$C_{\text{fs}}^{\text{Lee}}=\frac{P_{\text{opt}}-P_{0}}{P_{\max\;}-P_{0}},$$

where *P*_0_ (*P*_max_) is the minimum (maximum) hold-down pressure in the *P*-*H* curve and *P*_opt_ is the optimal hold-down pressure at which the pulse strength becomes maximal (see Figure 2). The *C*_fs_ in (1) varies within [0,1], and the pulse is said to be floating-like if *C*_fs_ is close to 0 and sunken-like if *C*_fs_ is close to 1. In this scheme, the hidden pulse can be considered naturally the limiting case of the sunken pulse (*C*_fs_ → 1). In a clinical test for determining the level of depth of the pulse by OMDs, Lee et al. showed that there was a significant difference in the averaged *C*_fs_ between subject groups diagnosed with floating pulse (for short, floating pulse group) and those with sunken pulse (for short, sunken pulse group), which verifies the *C*_fs_ method [19].

The *C*_fs_ defined in (1) needs an interpolation technique since the pulse is recorded over a few discrete steps of the hold-down pressure. When the measured pulse data do not follow an ideal pattern of smooth down-hill after up-hill with increasing hold-down pressure, or when the pressure steps are too few that the data points are not sufficient for a proper interpolation, the calculated *C*_fs_ may yield an erroneous correspondence to the level of pulse depth. An example of such an erroneous decision by the *C*_fs_^Lee^ method is illustrated in Figure 2(b), through which it is determined as a sunken-like pulse but an OMD would not diagnose it as a floating pulse or sunken pulse.

**Figure 3:**
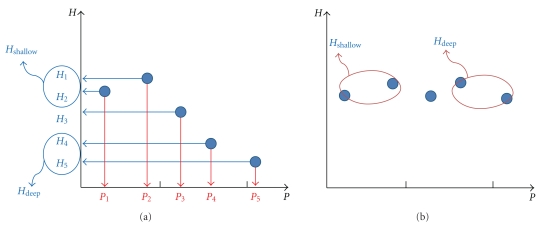
New algorithm for *C*_fs_ [20]. It compares explicitly the pulse strengths at low hold-down pressure and high hold-down pressure. Example for (a) a floating pulse, (b) a middle-depth pulse (neither floating nor sunken). Compare (b) with Figure 2(b).

Recently, Kim et al. proposed a new method to determine the pulse qualities related to the level of pulse depth, by explicit comparison of pulse strengths at a low hold-down pressure (*P*_shallow_) and at a high hold-down pressure (*P*_deep_) [20]. This method determines the pulse as a floating-like pulse if the pulse strength at low applied pressure is stronger than that at high hold-down pressure, and a sunken-like pulse if the pulse strength shows opposite behavior. This new proposal is more robust against anomalous data than the interpolated *P*-*H* curve method proposed by Lee et al., an example is shown in Figure 3(b).

By updating the proposal by Kim et al., we introduce a normalization constant as the sum of the pulse strengths, and define the new *C*_fs_ as

(2) $$C_{\text{fs}}^{\text{new}}=\frac{1}{2}\left(1+\frac{H_{\text{deep}}-H_{\text{shallow}}}{H_{\text{deep}}+H_{\text{shallow}}}\right)=\frac{H_{\text{deep}}}{H_{\text{deep}}+H_{\text{shallow}}},$$

where *H*_shallow_ (*H*_deep_) is the pulse strength at the pressure step of *P*_shallow_ (*P*_deep_). In Figure 3, it shows an appropriate definitions for *H*_shallow_ and *H*_deep_, given by *H*_shallow_ = (*H*_1_ + *H*_2_)/2 and *H*_deep_ = (*H*_4_ + *H*_5_)/2. Similar to the *C*_fs_^Lee^ by Lee et al., the new *C*_fs_ in (2) varies within [0,1], and the pulse is floating-like if *C*_fs_^new^ is close to 0 and sunken-like if *C*_fs_^new^ is close to 1. For equal pulse strength of *H*_shallow_ = *H*_deep_, the coefficient becomes *C*_fs_^new^ = 1/2. This method simulates OMDs who diagnose the pulse by comparing pulse strengths at two distinct pressure steps.

An appropriate assignment of pulse strengths *H*_shallow_ and *H*_deep_ depends directly on the properties of the pressure-steps applied in the pulse-taking device. The pulse-taking device used in our study applied hold-down pressure in five different steps, which were *P*_1_ = 37 ± 6, *P*_2_ = 73 ± 10, *P*_3_ = 108 ± 13, *P*_4_ = 143 ± 17, and *P*_5_ = 181 ± 21 mmHg on average (±standard deviation) over the subjects and over the palpation positions, for whom participated in this study. We found two appropriate choices for *H*_shallow_ and *H*_deep_ which showed best concordance with OMD decisions for the floating and sunken pulses (detailed comparisons between different choices of *H*_shallow_ and *H*_deep_ will not be addressed here); (1) (*H*_shallow_, *H*_deep_) = ((*H*_1_ + *H*_2_)/2, (*H*_4_ + *H*_5_)/2) and (2) (*H*_shallow_, *H*_deep_) = (*H*_1_, *H*_4_). Let us distinguish these two scenarios by labeling their coefficients of the floating and sunken pulses as *C*_fs_^new^(1) for (*H*_shallow_, *H*_deep_) = ((*H*_1_ + *H*_2_)/2, (*H*_4_ + *H*_5_)/2) and *C*_fs_^new^(2) for (*H*_shallow_, *H*_deep_) = (*H*_1_, *H*_4_). We will discuss the implications of these choices for (*H*_shallow_, *H*_deep_) in Discussion and Conclusions (Section 5).

The method using the *C*_fs_^new^ in (2) can be applied to simultaneous palpation by extending *H*_shallow_ and *H*_deep_ to be averaged all over Chon, Gwan, and Cheok. For example, for *C*_fs_^new^(2), the pulse strengths can be extended by (*H*_shallow_, *H*_deep_) = ((*H*_11_ + *H*_21_ + *H*_31_)/3, (*H*_14_ + *H*_24_ + *H*_34_)/3) and similarly for *C*_fs_^new^(1), where *H*_*ij*_ stands for the pulse strength at the *j*th pressure-step at Chon for *i* = 1, at Gwan for *i* = 2, and at Cheok for *i* = 3. In our study, as conventionally done in clinics to diagnose the pulse, OMDs palpated simultaneously at Chon, Gwan, and Cheok of the left wrist of each subject. For this reason, we adopt the *C*_fs_^new^ method with extended form of *H*_shallow_ and *H*_deep_ over Chon, Gwan, and Cheok.

## 3. Subjects and Methods

### 3.1. Subjects

Subjects who were simultaneously pulse-diagnosed by paired OMDs were composed of 169 healthy volunteers in their 20s with no vascular deformity on the radial artery. The purpose and protocol of the study were explained to the subjects prior to study commencement. The study was carried out with the approval of the Institutional Review Board of the Korea Institute of oriental Medicine (IRB, I0903-01-02). The basic health conditions of each subject were examined by the OMDs, based on listening and smelling, inquiry, inspection, and palpation, with the help of questionnaire and blood pressure measurement. The basic physiological data of the subjects are summarized in Table 1.

### 3.2. Study Design

**Figure 4:**
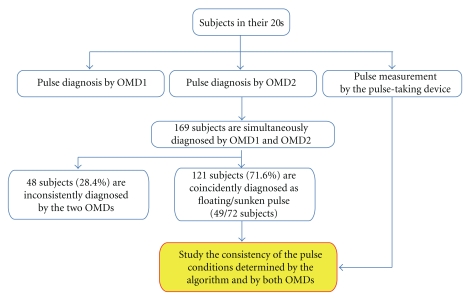
Study design and the outcomes of OMDs' pulse diagnoses.

The overall flow of the clinical study is illustrated in Figure 4. We attempted to reduce experiential bias of individual OMD by allocating pairs of OMDs to each subject. Each pair of OMDs was cyclically chosen from an OMD pool comprised of 12 practitioners with more than 5 years of clinical experience. OMDs diagnosed the pulse by simultaneously palpating at three locations on the left wrist-Chon, Gwan and Cheok. OMDs examined the pulses only on the left wrist to reduce degrees of freedom in case that the left and right pulses differ in their characteristics. For the 121 subjects who were concordantly diagnosed as floating or sunken pulses by paired OMDs, we analyzed the measured pulses by the pulse-taking device.

### 3.3. Pulse Measurement

The pulse was measured by 3-D MAC (Daeyomedi, Korea) approved by the Korea Food and Drug Administration. The 3-D MAC is equipped with an array-sensor with its elements arrayed crosswise within 10 × 10 mm^2^. Each sensing element is a piezoresistive pressure sensor of about 2 × 3 mm^2^. 

**Figure 5:**
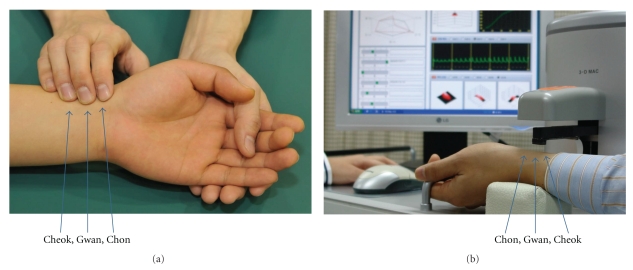
(a) palpation positions of Chon, Gwan, and Cheok, and (b) a demonstration of pulse-taking operation by the 3-D MAC.

A trained operator located the sensor at Gwan and initiated the measurement program. Before commencing actual pulse measurement, the device calibrated sensing performance and carried out fine adjustment to enable the central sensing element to locate at the center of the radial artery by automatically scanning nearby areas with varying hold-down pressures. It subsequently commenced measuring the pulse. The pulse was measured with applied hold-down pressures in five different steps. The applied pressure at each step was maintained constant for five seconds. For our study, the hold-down pressure at each pressure-step was set at 37  ±  6, 73  ±  10, 108  ±  13, 143  ±  17, and 181  ±  21 mmHg on average (±standard deviation). After measuring the pulse signal at Gwan, by an automated algorithm, the 3-D MAC shifted its sensor towards Cheok position and repeated measurement and subsequently it moved towards Chon position to complete the measurement. The 3-D MAC estimates that Cheok and Chon are about 10 mm away from Gwan which are comparable to the average palpation positions by OMDs for the pulse diagnosis (see Figure 5) [21]. We note that the way the 3-D MAC obtains pulse signals at Chon, Gwan, and Cheok is similar to the method of feeling the pulse with a single finger. The appearance of the 3-D MAC with its operational demo is shown in Figure 5(b). The performance of pulse measurement by the 3-D MAC is in the acceptable range of repeatability with the covariance for the maximum pulse strength at about 10%. 

### 3.4. Acquisition Process for the Pulse Strength for Each Pressure-Step

**Figure 6:**
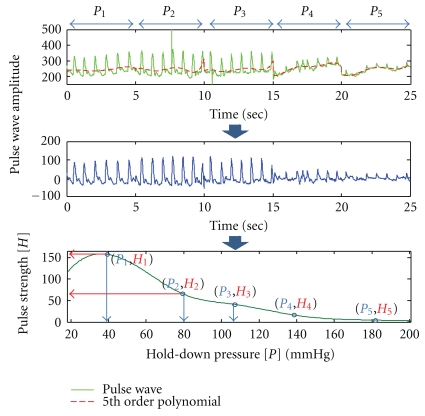
Data acquisition process from raw data (top panel) to the pulse strength for each pressure-step (bottom panel).

As shown in Figure 6 (top panel), raw data of the pulse waveform obtained from the measuring sensor contained noises due to breathing, uncontrolled movement of subject's arm, and so forth. Baseline alignment was done by the 5th-order polynomial approximation, yielding the middle panel. By obtaining the average waveform for each pressure-step, we finally obtain the pulse strength (*H*_1_ to *H*_5_) for each pressure-step (*P*_1_ to *P*_5_) as shown in the bottom panel in Figure 6. By making appropriate choices for *H*_shallow_ and *H*_deep_, we proceeded to calculate *C*_fs_^new^(1) and *C*_fs_^new^(2).

## 4. Results

### 4.1. Diagnosis Result by Paired OMDs

Paired OMDs diagnosed 169 subjects simultaneously either as floating or sunken pulses. Among them, the diagnoses on 121 subjects (71.6%) were concordant, while the diagnoses of the remaining 48 subjects (28.4%) were divergent. Among the concordant cases, 49 subjects (40.5%) were diagnosed with floating pulses, and 72 subjects (59.5%) with sunken pulses. 

Table 2 shows that the accuracy (alternatively, concordant diganoses) between OMD1 and OMD2 was 71.6% (121 agreements/169 simultaneous diagnoses), and the Matthews correlation coefficient (MCC) was 0.42. Note that Table 2 was obtained not by any fixed pairs of OMDs, but rather by cyclically paired OMDs among 12 OMD participants on each day of study. With these facts in mind, an accuracy above 70% and MCC above 0.4 are indicative of a good concordance between OMDs diagnoses.

### 4.2. Characteristics of the Floating Pulse Group and Sunken Pulse Group

We assumed diagnoses by paired OMDs returned reliable concordance. For the 121 subjects who were concordantly diagnosed with floating or sunken pulses by paired OMDs, we tabulate some physiological quantities stratified by pulse quality and gender in Table 3.

Table 3 shows that BMI and *C*_fs_^new^(1) and *C*_fs_^new^(2) were significantly different between the two pulse groups and in both genders at the significance level of *P* = .05, while blood pressures, heart rate, or *H*_max_ remained not different significantly. Here *H*_max_ is the maximum value among the measured pulse strengths ranging from *H*_1_ to *H*_5_, where *H*_*i*_ = (*H*_1*i*_ + *H*_2*i*_ + *H*_3*i*_)/3 is the pulse strength at the *i*th pressure-step averaged over the three palpation locations. Other parameters such as diastolic blood pressure, difference between systolic and diastolic blood pressure were not significantly different between the floating pulse group and sunken pulse group.

The Pearson correlation coefficient (PCC) between *C*_fs_^new^(1) and *C*_fs_^new^(2) for the 121 concordant subjects is 0.96. Despite strong correlation between *C*_fs_^new^(1) and *C*_fs_^new^(2), Table 3 shows that *C*_fs_^new^(2) is more meaningfully different than *C*_fs_^new^(1) between the floating and sunken pulse groups in both genders. It implies that *C*_fs_^new^(2) may give improved performance over *C*_fs_^new^(1) in predicting the floating and sunken pulses. Based on this observation, we examined the diagnostic performance of pulse qualities using *C*_fs_^new^(1) and *C*_fs_^new^(2), with reference to the OMDs' diagnoses. The performance of the algorithm with reference to the OMDs' diagnoses is slightly better in male volunteers than in female volunteers. Since the difference between two gender groups is minor, hereafter we do not distinguish genders and present results only for the entire subject group.

### 4.3. Determination of Pulse Qualities by *C*_fs_^new^(1) and *C*_fs_^new^(2)

**Figure 7:**
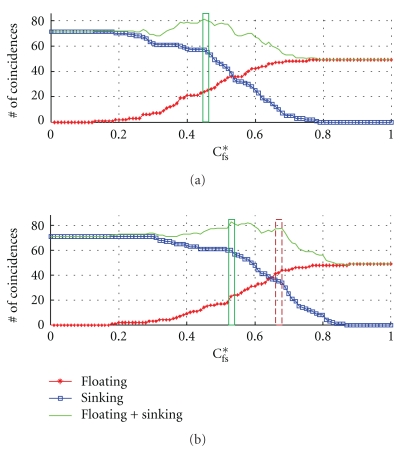
The number of decisions by (3) that are concordant with the diagnoses by paired OMDs, for the floating (asterisked curve) and sunken pulses (squared curve), as a function of the discriminant *C*_fs_*. Result by using *C*_fs_^new^(1) is shown in (a) and *C*_fs_^new^(2) in (b), where the plain curve corresponds to the total concordant decisions as the floating or sunken pulses, between the algorithm and OMDs. The boxed area in (a) is the regime showing both the maximal accuracy and MCC, while in (b) the accuracy is shown maximal in the solid box and the MCC is shown maximal in the dotted box.

We attempt to classify the measured pulses into the floating or sunken pulses using *C*_fs_^new^(1) and *C*_fs_^new^(2). A simple decision rule can be made by

(3) $$\begin{array}{cc} & \text{[If}\;C_{\text{fs}}\leq C_{\text{fs}}^{*},\;\text{then}\;\text{it}\;\text{is}\;\text{classified}\;\text{as}\;\text{a}\;\text{floating}\;\text{pulse},\\ & \;\;\;\text{else}\;\text{if}\;C_{\text{fs}}>C_{\text{fs}}^{*},\;\text{then}\;\text{it}\;\text{is}\;\text{classified}\;\text{as}\;\text{a}\;\text{sunken}\;\text{pulse]}, \end{array}$$

where *C*_fs_* is the discriminant for the floating and sunken pulses. Applying this rule, we obtain Figure 7.

Figure 7 shows the number of concordant decisions between the classification by the rule in (3) and diagnosis by paired OMDs as a function of the discriminant *C*_fs_*. Results by using *C*_fs_^new^(1) is shown in (a) and *C*_fs_^new^(2) in (b). As plotted by the asterisked curve, the number of concordant decisions for the floating pulse increase with increasing *C*_fs_*, while it was the opposite for the sunken pulse (squared curve). The overall maximum in the concordant decisions for the floating and sunken pulses lies in the central regime of *C*_fs_*. Specifically, the accuracy (ACC), which is the ratio between the concordant decisions and total decisions, is maximally given by ACC = 82/121 = 67.8% at *C*_fs_* = 0.45 with *C*_fs_^new^(1) (boxed region in (a)), and ACC = 84/121 = 69.4% at *C*_fs_* = 0.53 with *C*_fs_^new^(2) (solid box in (b)). On the other hand, the Matthews correlation coefficient (MCC) is maximally given by MCC = 0.31 at *C*_fs_* = 0.45 with *C*_fs_^new^(1) (boxed region in (a)), and MCC = 0.40 at *C*_fs_* = 0.68 with *C*_fs_^new^(1) (dotted box in (b)).

Decisions by both *C*_fs_^new^(1) and *C*_fs_^new^(2) show similar patterns in accuracy with maximum values given similarly, that is, ACC = 67.8% with *C*_fs_^new^(1) and ACC = 69.4% with *C*_fs_^new^(2). However, compared to *C*_fs_^new^(1), *C*_fs_^new^(2) shows improved correlations with decisions by OMDs. Noting that the MCC between pairs of OMDs is 0.42, the MCC between diagnoses by *C*_fs_^new^(2) and OMDs is reasonably good. Both the Matthews correlation coefficient and the two-sample *T*-test in Table 3 indicate that *C*_fs_^new^(2) yields better concordance with the pulse determinations by OMDs than *C*_fs_^new^(1). It implies that an adequate pair of the low and high applied pressures to discriminate the floating pulse from the sunken pulse is given by (*P*_shallow_, *P*_deep_) = (37 mmHg, 143 mmHg). We will discuss this result further in the section of Discussion and Conclusions.

**Figure 8:**
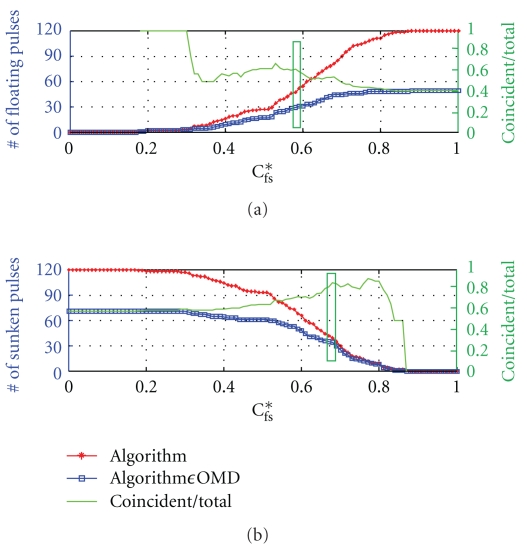
Decision by (4) with *C*_fs_^new^(2) for (a) the floating pulse and (b) sunken pulse as a function of the discriminant (a) *C*_f_* and (b) *C*_s_*. The asterisked curve corresponds to the total number of decisions, and the squared curve the concordant decisions with the OMDs (scale on the left vertical axis), while the plain curve is the accuracy (scale on the right vertical axis).

By setting the discriminant for the floating and sunken pulses independently, we can improve the accuracy with the regime between the two discriminants left undetermined. In this case, the Matthews correlation coefficient is poorly defined and we do not take this correlation into account. We denote the discriminant for the floating pulse by *C*_f_* and that for the sunken pulse by *C*_s_*. Then, the classification rule (3) is modified as

(4) $$\begin{array}{cc} & \text{[If}\;C_{\text{fs}}\leq C_{\text{f}}^{*},\;\text{then}\;\text{it}\;\text{belongs}\;\text{to}\;\text{the}\;\text{floating}\;\text{pulse}\;\text{group},\\ & \;\;\;\text{while}\;\text{if}\;C_{\text{fs}}>C_{\text{s}}^{*},\text{then}\;\text{it}\;\text{belongs}\;\text{to}\;\text{the}\;\text{sunken}\;\text{pulse}\\ & \;\;\;\text{group]}, \end{array}$$

where the determinants satisfy *C*_f_* ≤ *C*_s_*, and it reduces to (3) in the case of *C*_f_* = *C*_s_*. The result is shown in Figure 8 for *C*_fs_^new^(2) (we skip the result for *C*_fs_^new^(1)). Since the rule in (4) may have the regime of *C*_f_* < *C*_fs_ ≤ *C*_s_* left undetermined, we introduce the selection rate (SR) to count the determined pulses either as floating or sunken pulses with respect to the total number of subjects to be determined, that is,

(5) $$\begin{array}{c} \text{SR}=\frac{\text{actual}\;\text{number}\;\text{of}\;\text{decisions}\;\text{made}}{\text{number}\;\text{of}\;\text{subjects}\;\text{to}\;\text{be}\;\text{determined}}. \end{array}$$

Figure 8 shows classifications into (a) the floating pulse and (b) the sunken pulse by the rule in (4) with *C*_fs_^new^(2). Both the total number of decisions (asterisked curve) and the concordant decisions with OMDs' (squared curve) increase with increasing *C*_f_* for the floating pulse, while they increase with decreasing *C*_s_* for the sunken pulse. On the other hand, the accuracy (plain curve) tends to increase with decreasing number of decisions made. The sudden drop in accuracy for the sunken pulse close to 1 (*C*_s_* ≥ 0.85) is unimportant anomaly that may occur with small sample size. An optimal pair of two discriminants turns out to be (*C*_f_*, *C*_s_*) = (0.58, 0.68) (boxed areas) with respective accuracy given by ACC = 29/47 = 61.7% for the floating pulse, and ACC = 35/40 = 87.5% for the sunken pulse. The overall accuracy for the 87 determined cases is ACC = 64/87 = 73.6%, which is improvement of about 4.2% compared to the result by (3), while 34 subjects (28.1%) remained undetermined as the opportunity cost. A similar result can be obtained with *C*_fs_^new^(1). For instance, for an optimal pair of (*C*_f_*, *C*_s_*) = (0.45, 0.52), ACC = 24/38 = 63.3% for the floating pulse and ACC = 43/57 = 75.4% for the sunken pulse. The overall accuracy for the 95 determined cases is ACC = 67/95 = 70.5%, which is improvement of about 2.7% in accuracy with 26 subjects (21.5%) left undetermined.

Depending on one's preference for accuracy versus selection rate, one can have different combinations for an optimal pair of discriminants (*C*_f_*, *C*_s_*). For instance, choosing *C*_s_* much larger than *C*_f_* is expected to increase the accuracy but decrease the selection rate, while choosing an equal value for *C*_s_* and *C*_f_* will maximize the selection rate (SR = 1) but at the expense of the accuracy. To examine gain and loss between the selection rate and accuracy as a function of the discriminants (*C*_f_*, *C*_s_*), we calculated the number of concordant decisions and the number of decisions made on two-dimensional space spanned by *C*_f_* and *C*_s_*.

**Figure 9:**
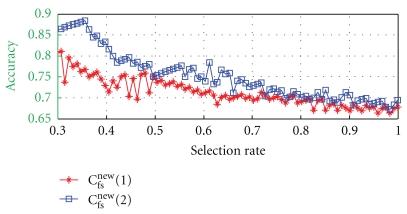
Decision by (4) with *C*_fs_^new^(1) (asterisked curve) and (b) *C*_fs_^new^(2) (squared curve). The graph shows the accuracy for the maximum number of coincidences with OMDs' decisions as a function of the decision rate, that is, the number of decisions made into the floating or sunken pulses.

As a result, in Figure 9, we present the accuracy for the maximum concordant decisions as a function of the selection rate, where the asterisked curve presents results by *C*_fs_^new^(1) and the squared curve presents results by *C*_fs_^new^(2). There exists an optimal pair of discriminants (*C*_f_*, *C*_s_*) with which the concordant decisions become maximal for a given number of decisions made as the floating or sunken pulses (refer Figure 8). Information on the optimal pairs of discriminants is not shown in the figure. It shows better accuracy with *C*_fs_^new^(2) than with *C*_fs_^new^(1) in the entire range of the selection rate. Especially, for *C*_fs_^new^(2), a meaningful improvement in accuracy is observed at the expense of selection rate.

Indeed, the undetermined regime of *C*_fs_ satisfying *C*_f_* < *C*_fs_ ≤ *C*_s_* can be classified into the group of middle-depth pulse. Then (4) can be modified to classify the pulse into the three groups in the level of depth by

(6) $$\begin{array}{cc} & \text{[If}\;C_{\text{fs}}\leq C_{\text{f}}^{*},\;\text{then}\;\text{it}\;\text{belongs}\;\text{to}\;\text{the}\;\text{floating}\;\text{pulse}\;\text{group},\\ & \;\;\text{else}\;\text{if}\;C_{\text{fs}}>C_{\text{s}}^{*},\text{then}\;\text{it}\;\text{belongs}\;\text{to}\;\text{the}\;\text{sunken}\;\text{pulse}\;\text{group},\\ & \;\;\text{otherwise}\;\left(C_{\text{f}}^{*}<C_{\text{fs}}\leq C_{\text{s}}^{*}\right)\;\text{it}\;\text{belongs}\;\text{to}\;\text{the}\;\text{middle-depth}\\ & \;\;\text{pulse}\;\text{group]}. \end{array}$$

Equation (6) classifies a pulse into a floating, a sunken, or a middle-depth pulse, which simulates OMDs clinical diagnosis in practice. Applying this rule to Figure 9, the number of the middle-depth pulse becomes “the number of subjects − number of decision” (reading *x*-axis). It shows a clear tendency for *C*_fs_^new^(2) (squared curve) that increasing the number of the decisions into the middle-depth pulse group increases the accuracy in the decisions into the floating and the sunken pulse groups.

## 5. Discussion and Conclusions

We proposed a novel classification algorithm for the floating pulse and sunken pulse and carried out a clinical test to examine its validity. First, we introduced the coefficient of the floating and sunken pulses (*C*_fs_) which is bounded between 0 and 1. When *C*_fs_ is close to 0, it is classified as a floating pulse, When *C*_fs_ is close to 1, it is classified as a sunken pulse. Using the classification algorithm based on the new *C*_fs_ method, we performed a clinical study to determine correlation between diagnoses made by the OMDs and by the algorithm. 

For the pulse decisions by OMDs, out of 12 OMD participants, we paired two OMDs in different combination on each day of the study. Among 169 simultaneous diagnoses made by paired OMDs, concordant diagnoses were made on 121 subjects (72%), and the rest (28%) showed divergent diagnoses, yielding a Matthews correlation coefficient of 0.42. It lies within a reasonable range of agreement between the OMDs. Out of the 121 concordant diagnoses, 72 were diagnosed as the sunken pulses (sunken pulse group), and 49 were the floating pulses (floating pulse group). 

Two-sample *T*-tests between the floating pulse group and the sunken pulse group showed that BMI and *C*_fs_ were significantly different (*P* < .05) in both genders. This result is in accordance with an experiential knowledge that obese individuals are likely to have sunken pulses [4]. We can assume that there is a positive correlation between BMI and skin thickness. This leads to the conclusion that a major factor affecting the pulse depth may be tissue thickness between the radial artery and skin surface. A complementary experiment, for example, measuring skin thickness by ultrasonography, will help us verify the assumed correlation. On the other hand, average blood pressure between the floating and sunken pulse groups was different at the significance level of *P* = .1, while it did not show differences in heart rate, maximum pulse strength, and so forth It indicates that average blood pressure is another factor which determines the pulse depth. By these observations, we suggest that two major factors determining the pulse depth are the thickness of skin tissue above the radial artery and the average blood pressure. 

For the 121 subjects diagnosed concordantly by paired OMDs, we tested for consistency with the novel classification algorithm. For the entire study cohort, the maximum accuracy reached was about 68% ~ 69% using coefficient *C*_fs_^new^(1) or *C*_fs_^new^(2), while the Matthews correlation coefficient reached was up to 0.40 for *C*_fs_^new^(1) and 0.31 for *C*_fs_^new^(1). It suggests that the classification algorithm using either coefficient yields an acceptable concordance with OMDs' diagnoses, and *C*_fs_^new^(2) yields an optimal performance in its concordance. It suggests that an adequate pair of low and high applied pressures for our algorithm is given by (*P*_shallow_, *P*_deep_) = (37 mmHg, 143 mmHg). Since our algorithm simulates diagnosis by OMDs who compare pulse strengths at low and high hold-down pressures to determine the pulse depth, the above result implies that OMDs are likely to determine the pulse depth by comparing pulse strengths at a weak applied pressure of *P*_shallow_ ≈ 37 mmHg and at a strong pressure of *P*_deep_ ≈ 143 mmHg, one of which lies much below the normal range of the blood pressure and the other one lies above. 

When the pulse strength is larger at *P*_shallow_ than at *P*_deep_, the maximal pulse strength will be positioned well below the average blood pressure (*P*_avg_ ≈ 85 mmHg) and it shows a typical floating characteristic, and when the pulse strength at *P*_deep_ is larger, it shows a sunken characteristic. On the other hand, if pulse strengths at *P*_shallow_ and *P*_deep_ are not so different, the maximum pulse will be observed around *P*_avg_, yielding a middle-depth pulse. A more accurate choice for the low and high pressures to be used in the algorithm can be made with a pulse-taking device that offers more narrowly spaced hold-down pressures. A pulse-taking device with 9 steps of the hold-down pressure that are spaced equally up to 180 mmHg is an appropriate candidate for such purpose.

Finally, we suggested an updated version of the above-tested algorithm by allowing a regime of the middle-depth pulse group for the pulses satisfying *C*_f_* < *C*_fs_ ≤ *C*_s_*, which is more useful since OMDs in clinics diagnose a pulse either as floating, sunken, or neither of them corresponding to the middle-depth pulse. The implication of the middle-depth pulse is a sign of balance between Yang/function (Qi) and Yin/form (blood) [3].

In conclusion, we proposed a novel algorithm to classify pulse into floating, sunken, or middle-depth categories using a coefficient called *C*_fs_. By a clinical test, primarily we confirmed the validity of the novel classification algorithm and secondly we found appropriate numerical values of the pressure steps which are apt for determining the level of pulse depth. To our knowledge, this study is the first report on developing classification algorithm for the floating and sunken pulses based on a massive clinical data with human subjects of the order of hundred. This work may motivate further research activities towards developing objective classification algorithms for other pulse qualities.

## Figures and Tables

**Table 1:** Subject information.

Characteristic (unit)	Value (mean ± SD)
Number (*n*)	169 (male = 88, female = 81)
Age (yr)	23.7 ± 2.4
Height (cm)	169.0 ± 8.2
Weight (kg)	63.6 ± 11.5
BMI (kg/m^2^)	22.1 ± 3.0
Systolic/diastolic blood pressure (mm Hg)	120.9/67.4 ± 16.2/11.2

**Table 2:** Consistency of the pulse diagnoses between paired OMDs.

		OMD1		
		Floating	Sunken	Total
OMD2	Floating	49	22	71
	Sunken	26	72	98
	Total	75	94	169

**Table 3:** Characteristics of 121 subjects stratified by pulse quality and gender.

SEX	Pulse (subject^#^)	BMI (kg/m^2^)	Bp_systole_ (mmHg)	*〈*Bp*〉* (mmHg)	Heart rate (beats/min)	*H*_max _	*C*_fs_^new^(1)	*C*_fs_^new^(2)
Male	Floating (26)	21.3 ± 1.6	126.0 ± 16.0	85.1 ± 11.6	80.4 ± 14.5	170.2 ± 40.1	0.46 ± 0.13	0.55 ± 0.13
	Sunken (32)	24.2 ± 3.2	129.4 ± 14.1	91.2 ± 10.8	77.1 ± 12.2	160.5 ± 30.6	0.58 ± 0.10	0.66 ± 0.09
	*T*-test (*P*-value)	1.2E-4**	0.38	0.044*	0.35	0.30	5.1E-4**	3.2E-4**

Female	Floating (23)	19.4 ± 2.0	109.0 ± 12.2	79.0 ± 7.2	83.0 ± 12.4	143.4 ± 33.5	0.43 ± 0.14	0.52 ± 0.16
	Sunken (40)	22.3 ± 2.8	115.3 ± 13.0	82.9 ± 9.9	79.9 ± 11.0	136.0 ± 35.3	0.49 ± 0.16	0.61 ± 0.16
	*T*-test (*P*-value)	6.2E-5**	0.063	0.10	0.30	0.42	0.12	0.029*

All	Floating (49)	20.4 ± 2.0	118.0 ± 16.6	82.2 ± 10.2	81.6 ± 13.5	157.6 ± 39.2	0.45 ± 0.14	0.54 ± 0.14
	Sunken (72)	23.1 ± 3.1	121.6 ± 15.2	86.6 ± 11.1	78.6 ± 11.5	146.9 ± 35.3	0.53 ± 0.14	0.63 ± 0.14
	*T*-test (*P*-value)	4.3E-7**	0.22	0.031*	0.20	0.12	1.8E-3**	1.9E-4**

Data presented are the mean ± SD except for the *T*-test result. *: *P* < .05, **: *P* < .005. Abbreviated: “Bp_systole_” = systolic blood pressure, “*〈*Bp*〉*” = average blood pressure, and “*H*_max _” stands for the maximum pulse strength among all pressure steps, whose unit is device-specific. “*T*-test” = the two-sample *T*-test between the floating and sunken pulse groups.
